# Framework of Methodology to Assess the Link between A Posteriori Dietary Patterns and Nutritional Adequacy: Application to Pregnancy

**DOI:** 10.3390/metabo12050395

**Published:** 2022-04-27

**Authors:** Foteini Tsakoumaki, Charikleia Kyrkou, Maria Fotiou, Aristea Dimitropoulou, Costas G. Biliaderis, Apostolos P. Athanasiadis, Georgios Menexes, Alexandra-Maria Michaelidou

**Affiliations:** 1 Department of Food Science and Technology, School of Agriculture, Faculty of Agriculture, Forestry and Natural Environment, Aristotle University of Thessaloniki, 541 24 Thessaloniki, Greece; foteinitsak@hotmail.com (F.T.); ckyrkou@hotmail.gr (C.K.); fotioum@yahoo.gr (M.F.); dimitropa@hotmail.com (A.D.); biliader@agro.auth.gr (C.G.B.); 2 3rd Department of Obstetrics and Gynecology, School of Medicine, Aristotle University of Thessaloniki, 541 24 Thessaloniki, Greece; apostolos3435@gmail.com; 3 Department of Field Crops and Ecology, School of Agriculture, Faculty of Agriculture, Forestry and Natural Environment, Aristotle University of Thessaloniki, 541 24 Thessaloniki, Greece; gmenexes@agro.auth.gr

**Keywords:** maternal nutrition, nutrient patterns, dietary quality, nutritional status, nutritional adequacy, principal component analysis, hierarchical cluster analysis, MedDiet Score, HEI-2010, dietary glycemic index

## Abstract

This study aimed to explore the nutritional profile of 608 women during the second trimester of pregnancy, in terms of nutrient patterns, dietary quality and nutritional adequacy. Dietary data were collected using a validated Mediterranean-oriented, culture-specific FFQ. Principal component analysis was performed on 18 energy-adjusted nutrients. Two main nutrient patterns, “plant-origin” (PLO) and “animal-origin” (ANO), were extracted. Six homogenous clusters (C) relative to nutrient patterns were obtained and analyzed through a multidimensional methodological approach. C1, C5 and C6 scored positively on PLO, while C1, C2 and C3 scored positively on ANO. When dietary quality was mapped on food choices and dietary indexes, C6 unveiled a group with a distinct image resembling the Mediterranean-type diet (MedDiet Score = 33.8). Although C1–C5 shared common dietary characteristics, their diet quality differed as reflected in the HEI-2010 (C1:79.7; C2:73.3; C3:70.9; C4:63.2; C5:76.6). The appraisal of nutritional adequacy mirrored a “nutritional-quality gradient”. A total of 50% of participants in C6 had almost 100% adequate magnesium intake, while 50% of participants in C4 had a probability of adequacy of ≤10%. Our methodological framework is efficient for assessing the link between a posteriori dietary patterns and nutritional adequacy during pregnancy. Given that macro- and micronutrient distributions may induce metabolic modifications of potential relevance to offspring’s health, public health strategies should be implemented.

## 1. Introduction

Pregnancy is an essential stage of the lifecycle, during which the in utero nutritional environment is sensitive to the habitual diet of the mother [1]. Indeed, this critical period of life is widely recognized as a key “window of opportunity” for linking early nutrition with healthy living, growth and development [2,3,4,5,6]. However, in the context of the global nutritional transition to increased consumption of energy-dense but nutrient-poor diets, nutritional inadequacies during pregnancy are a common challenge even for the developed world [2,4,7,8,9,10]. Therefore, the assessment of dietary patterns and diet quality may contribute to the development of public health strategies and preventive actions [11].

Indeed, dietary pattern analysis may serve as a valuable tool in exploring the potential interactions and cumulative effects of food intake on pregnancy outcomes [7,12,13,14,15,16,17]. Pattern analysis can be conducted using the hypothesis-oriented or the data-oriented approach. The hypothesis-oriented approach, also known as the a priori method, entails the use of dietary quality indexes that measure adherence to a predefined dietary pattern by exploring the balance between “beneficial” and “non-beneficial” per case components. On the other hand, the data-oriented approach, or the a posteriori method, is an empirically driven method summarizing the dietary intake of a population into dietary or nutrient patterns, through statistical modeling techniques [7,18,19,20,21]. In fact, the a posteriori approach contributes to unraveling the intrinsic complexity emerging from dietary data and displays the unique features of diet which may not be captured by any predefined score [7,22]. At a more immediate level, a posteriori dietary patterns provide insight into dietary behaviors mirroring actual food or nutrient intake [7].

Scientific data offer sufficient evidence in this exciting aspect of public health and nutritional epidemiology [14,15,18,23,24,25,26] since there is a cycle of passing “health capital” from one generation to the next [9]. However, to the best of our knowledge, the link between a posteriori dietary patterns and nutritional adequacy, at least during pregnancy, is rather limited to food-based patterns [27,28] and has not been extensively documented in the literature. Therefore, the aim of the present study was to explore the nutritional profile of pregnant women, in a Mediterranean country, Greece. For the realization of this project, the following sub-goals were targeted: (a) identification of a posteriori nutrient patterns using principal component analysis (PCA); (b) definition of clusters related to these patterns; (c) evaluation of clusters’ dietary quality, in terms of food consumption and a priori dietary indexes; (d) appraisal of clusters’ nutritional adequacy levels.

## 2. Results

### 2.1. Identification of Nutrient Patterns

Principal component analysis was performed on 18 energy-adjusted nutrients, and 2 main nutrient patterns (factors) were retained, explaining 28.4% and 27.3% of the total variance. The Kaiser–Meyer–Olkin (KMO) value was 0.785, verifying the good sampling adequacy for the analysis, and Bartlett’s test was <0.001, indicating that the 18 nutrient variables were correlated sufficiently for PCA to be conducted. The two extracted factors were labeled according to the typical origin of the nutrients with higher loadings within each factor, namely, the “plant-origin” factor and the “animal-origin” factor (Table 1).

The “plant-origin” factor was structured by folate, magnesium, potassium, thiamin, vitamin B-6, copper, niacin and vitamin C. As shown in Table 1, the carbohydrates/fiber variable had an opposite loading sign. The “animal-origin” factor consisted of phosphorus, vitamin B-12, animal protein/plant protein, calcium, riboflavin, zinc, selenium and cholesterol. The unsaturated (monounsaturated plus polyunsaturated)-to-saturated fatty acid ratio ((MUFA + PUFA)/SFA) variable was negatively loaded on this factor.

### 2.2. Homogenous Groups of Participants

Hierarchical cluster analysis (HCA) was performed on the scores of the two factors and revealed a six-group interpretable and statistically significant clustering of participants. A total of 179 participants (29.4%) were in Cluster (C) 1, 33 (5.4%) in C2, 142 (23.4%) in C3, 67 (11.0%) in C4, 127 (20.9%) in C5 and 60 (9.9%) in C6. Both “plant-origin” and “animal-origin” factors contributed almost equally to the formation of the clusters (R^2^ = 0.681, *p* < 0.001, and R^2^ = 0.719, *p* < 0.001, respectively).

### 2.3. Evaluation of Clusters’ Profile—A Multidimensional Approach

The clusters’ profile was analyzed through a multidimensional approach.

#### 2.3.1. First-level Approach—Mean Nutrient Patterns’ Scores

**Figure 1:**
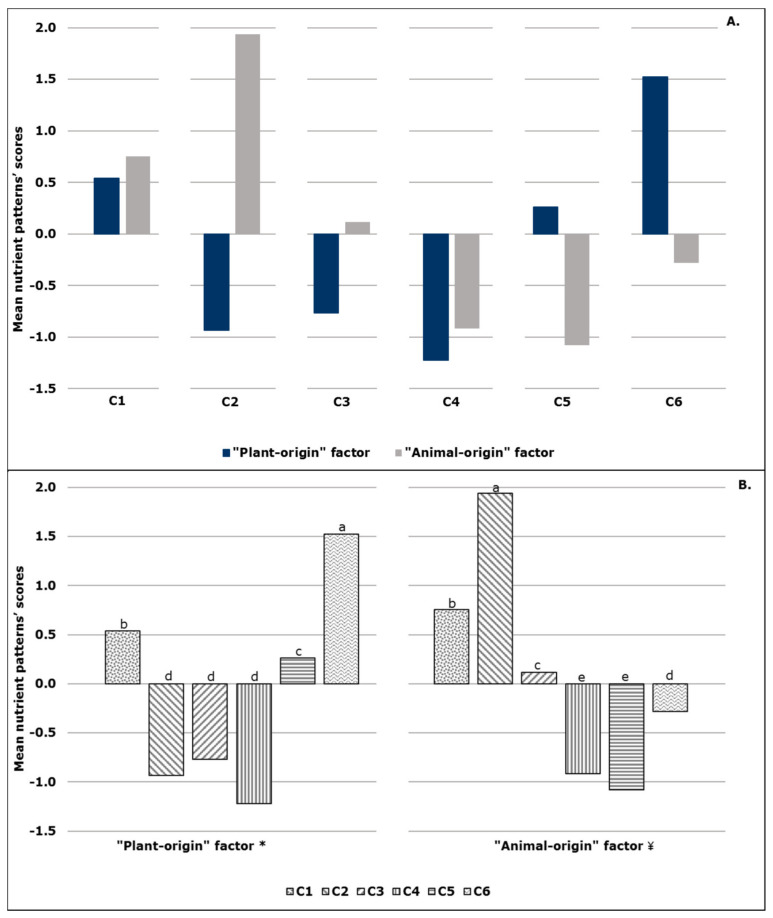
Mean nutrient patterns’ scores of “plant−origin” and “animal−origin” factors within (**A**) and between (**B**) clusters (C1−C6). Different superscript letters over bars represent statistical differences between clusters at *p* < 0.05; * according to Tukey’s test; ¥ according to the Games−Howell test.

The mean nutrient patterns’ scores of the six clusters are graphically illustrated in Figure 1A. C1 had positive mean scores, both on the “plant-origin” and the “animal-origin” factor, in contrast to C4, which demonstrated negative scores on both factors. As it is apparent from the visual inspection of Supplementary Figures S1 and S2, these findings could be, in part, attributed to the “position” of the mean intake value of a specific nutrient in each cluster, relative to the respective mean value in the total sample. For instance, participants in C1 had, compared to the mean value of the total sample, higher mean intakes for all nutrients that loaded positively on the two factors, except for the carbohydrates/fiber and (MUFA + PUFA)/SFA ratios that loaded negatively on the “plant-origin” and the “animal-origin” factor, respectively.

Through the application of analysis of variance, as shown in Figure 1B, C6 had the highest mean value in “plant-origin” factor scores (*p* < 0.001), while C2–C4 had the lowest mean values. Regarding the “animal-origin” factor, the highest mean value was observed in C2 (*p* < 0.001), whereas the lowest was observed in the cases of C4 and C5 (*p* < 0.001).

#### 2.3.2. Second-Level Approach—Demographic/Anthropometric and Lifestyle Characteristics

Table 2 shows the demographic/anthropometric and lifestyle characteristics of the participants in each cluster. No differences were recorded for maternal age, pre-pregnancy body mass index (BMI), education and physical activity level among clusters. Given that the majority of the pregnant women were non-smokers, a borderline important association was observed between smoking during pregnancy and the six dietary clusters.

#### 2.3.3. Third-Level Approach—Food Consumption and Dietary Indexes

**Figure 2:**
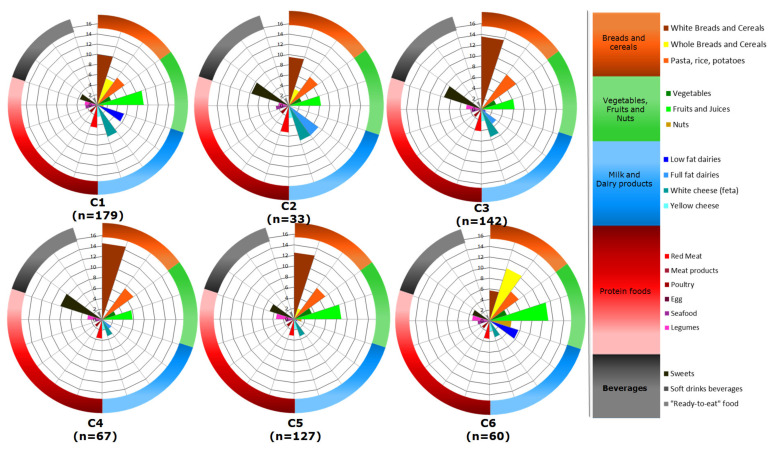
Schematic representation of the percentages of energy contributions of selected food groups, expressed as median values, in the six clusters (C1–C6).

The energy contribution of selected food groups in the six clusters is sketched in Figure 2. Each radar chart depicts the median values of the 19 food groups under consideration in each cluster. As such, a brief description of these plots will facilitate the identification of the prominent features in each case.

The consumption of “white breads and cereals” differed among clusters, with the minimum level, approximately equal to 6%, recorded for C6. On the contrary, C6 exhibited the highest consumption of “whole breads and cereals”, while the respective consumption value of this food group was equal to 0% for C3 and C4. The latter observation implies that 50% of C3 and C4, i.e., 71 and 33 participants, respectively, did not consume any whole-grain products. Another point worth commenting on is that 50% of the participants in C2, C3 and C4 consumed only “full-fat” dairy, while the respective proportion of women in C1 and C6 only consumed “low-fat” dairy. It is also important to note that the median value for both “full-fat” and “low-fat” dairy in C5 was almost 0%, indicating that 64 participants did not consume any milk and yogurt, regardless of the fat content. The pronounced preference of the 50% of participants in C2, C3 and C4 for the “sweets” group is also worthy of attention, as evidenced in Figure 2.

**Figure 3:**
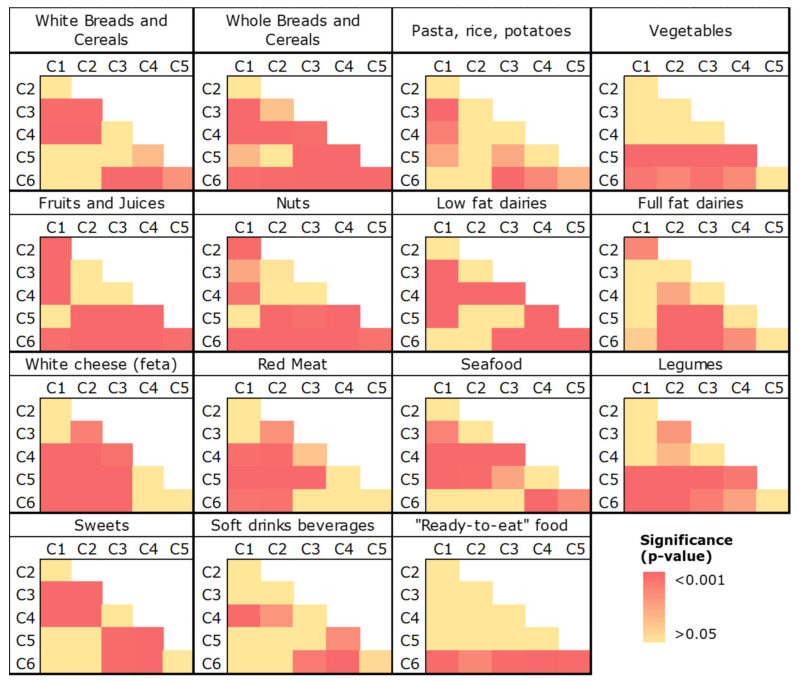
Comparison of energy contributions of selected food groups among the six clusters (C1–C6). Statistical significance was assessed at *α* = 0.05 (*p* ≤ 0.05) using the Mann–Whitney U test.

These initial observations were confirmed by the non-parametric comparison of the distributions (Table 3, Figure 3, Supplementary Figure S3). Thus, data are presented not only as median values, but also as measures of variability and, specifically, as the 25th and 75th percentiles, referred to as the interquartile range (IQR) (Table 3).

As depicted in Figure 3 and in accordance with the aforementioned findings, the energy contribution from “whole breads and cereals”, “fruits and juices” and “nuts” was higher in C6 compared to the other five clusters (Table 3, Figure 3). The energy contribution of “vegetables” and “legumes” did not differ between C5 and C6 and was significantly lower in C1–C4. On the other hand, participants in C6 reported a lower consumption of “red meat” (median: 3.4%, IQR: 2.5–4.8%), compared to C1 and C2 (median: 4.5%, IQR: 3.2–6.1%, and median: 5.3%, IQR:3.4–7.7%, respectively, Table 3, Figure 3). The consumption of “sweets” in C6 was also low compared to that in other groups, i.e., C3 and C4. The aforementioned features of C6 led us to assume that this dietary pattern had the highest nutritional quality under the conditions of this study. As expected, this was confirmed by the application of the a priori approach, since C6 had the greatest mean values of the MedDiet Score and Healthy Eating Index (HEI)-2010, and simultaneously the lowest mean value of the dietary glycemic index (GI) (Table 4).

From the data in Table 3 and Figure 3, it can be found that the poorest diet quality profile was reflected in the dietary behavior of participants in C4. This assumption can be attributed to the high consumption of moderate components, such as refined grains and empty calories (sweets and soft drink beverages). As such, the MedDiet Score (28.7 ± 3.2) and the HEI-2010 mean value (63.2 ± 8.0) in C4 were statistically significantly lower compared to the other clusters, while the estimated dietary GI was the highest (78.5 ± 4.2, Table 4).

#### 2.3.4. Fourth-Level Approach—Nutritional Adequacy

**Figure 4:**
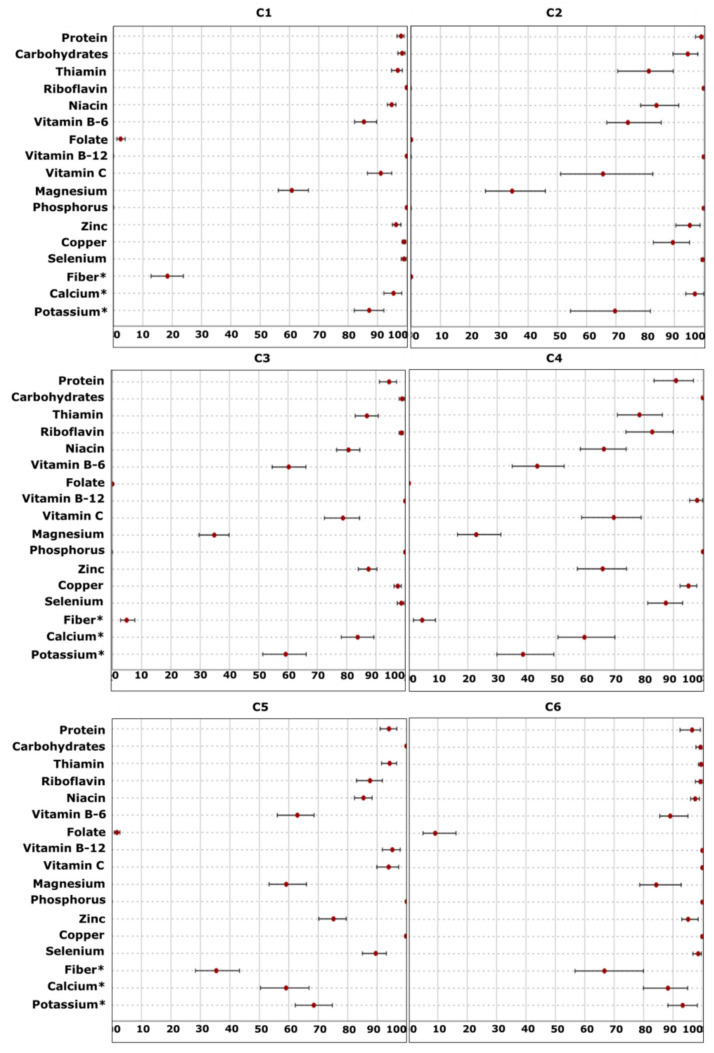
Point estimates and bootstrap confidence intervals (BCa CI) for the nutritional adequacy (%) in each cluster (C). Nutritional adequacy was assessed with the probability approach. For nutrients with *, adequacy was estimated with the EAR cut-point method.

The point and interval estimates (95% bias-corrected and accelerated bootstrap confidence intervals, BCas CI) for the nutritional adequacy in each cluster are presented in Figure 4. The most striking points emerging from Figure 4 relate to: (a) the high degree of nutritional adequacy (>90%) that was achieved in all clusters for protein, carbohydrates, vitamin B-12, phosphorus and copper; (b) the low level of folate adequacy that was recorded in all clusters; (c) the overall low to moderate degree of adequacy in C4; (d) the overall higher nutritional adequacy of C6 compared to the other clusters.

In order to unravel the extent of nutritional adequacy, the percentile distribution of the probability of adequacy is provided in Table 5 and Supplementary Table S1. For nutrients with an established AI, i.e., fiber and potassium, or without a standard deviation (SD) of requirement, i.e., calcium, the detailed descriptive statistics are presented in Table 6.

##### Probability of Adequacy

The probability of adequacy recorded for magnesium in C6 ranged from 34% (*P*10) to 100% (*P*90) (Table 5). However, even from the *P*50, participants had almost 100% adequate intakes. By contrast, the extent of adequacy in C4 ranged from 0% (*P*10) to 79% (*P*90), while 50% of the participants (≤*P*50) were identified as a high-risk group, since the level of adequacy at *P*50 was 10%. A low level of adequacy was also observed at *P*50 in C3 (17%).

The percentile distribution of adequacy in the case of vitamin B-6 was also indicative of the different nutritional status of the participants. A total of 50% of the participants (≥*P*50) in C1 and C6 had almost 100% adequate vitamin B-6 intake. On the other hand, this probability in C4 was equal to 35%, while the adequacy obtained at *P*10 unraveled “hidden” high-risk groups of participants (*P*10: 2%). 

##### EAR Cut-Point Method

Differences in the level of adequacy were also recorded when the EAR cut-point method was applied for fiber, potassium and calcium (Table 6). In the case of fiber, it is apparent from Table 6 that C2–C4 had the lowest level of adequacy (0–4.9%), since individuals in these clusters did not meet the adequate intake (AI) value (28 g/d) even at *P*90. In contrast, in C6, the AI was met between *P*25 and *P*50, and therefore a higher level of adequacy was observed (66.7%). A high level of adequacy was also observed in C6 for potassium (93.3%). In fact, the AI value (2.9 g/d) was met at *P*10. As it is also presented, different levels of adequacy were also recorded in the case of calcium. In fact, participants in C1 and C2 demonstrated a high level of adequacy (>95%), while in C4 and C5, a moderate level was recorded, approximately equal to 59%.

## 3. Discussion

In the present study, we assessed the nutritional profile of 608 pregnant women in a Mediterranean country, Greece. Two nutrient patterns labeled as “plant-origin” and “animal-origin” factors were extracted using PCA. The application of HCA to individuals’ factor scores classified the participants into six distinct clusters. Our framework of the methodology proved to be efficient and useful for assessing the link between a posteriori nutrient-based patterns and nutritional adequacy during pregnancy.

### 3.1. Commentary on Issues of Importance in This Study

#### 3.1.1. Methodological Design

Exploratory factor analysis is a statistical method that allows for deriving empirical dietary patterns and has been extensively employed as a tool for the assessment of maternal diet during pregnancy [7,23,24,26,30,31,32,33]. This data-driven technique reduces the dimensionality of dietary variables to a few components [34]; nevertheless, the natural interpretability of the derived factors depends on several subjective and arbitrary decisions. The key aspects of these could be examined under the following two headings: (a) the input dietary variables and their preprocessing, and (b) the statistical criteria for model selection [13,22,35,36,37,38,39].

As far as the input dietary variables are concerned, Lovegrove et al. [20] suggested that food items, food groups, nutrients or combinations can be used in all exploratory methods. Although extensive research has been carried out on the identification of maternal food-based patterns [23,24,26], the nutrient-based patterns are not very common [7]. Foods may indeed be easier to translate into public health recommendations [22], but a “nutrient approach”, mirroring synergic effects and interactions [22,40], may provide valuable information regarding the potential underlying mechanisms that link maternal diet and offspring health [7]. Furthermore, nutrients are universal and independent of sociocultural and geographic influences, facilitating comparisons between populations [22,36,41,42]. Towards this direction, the first step in our methodology was to generate nutrient patterns. For the entry of variables into the factor analysis, and consistent with previous studies [7,22,43], we chose to consider energy-adjusted nutrient intakes, to mitigate the dominating effect of those nutrients with the largest variance.

In terms of the statistical criteria used for model selection, the proportion of the total variance explained by our two retained factors was comparable to that recorded in previous studies on nutrient patterns [7,40,44]. Furthermore, similar factor structures and nomenclatures have been reported in the literature [36,40,42,44].

#### 3.1.2. Dietary Patterns and Nutritional Adequacy

To the best of our knowledge, research on maternal nutrient-based patterns is limited. Therefore, the critical assessment of our results was performed against published data obtained by maternal food-based patterns.

Participants in C6 tended to create a group with distinct dietary features—higher contribution from core foods of the Greek cuisine—and a distinct image resampling of the “Mediterranean”-, “Health Conscious”- or “Prudent”-type diet [27,30,31,39,45,46,47,48]; however, the assessment of dietary behavior became more complicated in C1–C5. Although, at first glance, women in C1–C5 shared common dietary characteristics with the previously identified patterns of the “Occidental”-, “Western”-, “Processed”- or “Unhealthy”-type diet [17,27,30,31,45,46,47,48], the diet quality differed among these clusters, as reflected overall in the a priori indexes calculated herein. The pronounced preference of participants in C3 and C4 for sweets and soft drink beverages was also in agreement with the “Snack-type” pattern identified in Greek adult women by Karageorgou et al. [49]. From a general perspective, our findings verify the evidence that adult populations in the Mediterranean region may exhibit healthier dietary behaviors even when some typical characteristics of a “Western”-type diet are evident [49,50]. As such, the mean value of HEI-2010 in C4 was higher than the threshold of 59, which represents a rather poor diet quality [51].

The potential of the a posteriori pattern analysis to assess the nutritional adequacy of pregnant women has been demonstrated in previous studies based on foods [27,28,30,52,53,54]. In accordance with our results, McGowan et al. [27] recorded greater nutritional adequacy among women in the “Health conscious” group compared to an “Unhealthy” pattern. In the same line, Grieger et al. [53] suggested that the adoption of a “high-fat/sugar/take-away” pattern, in the preconception period, is likely to reflect a poor nutritional status during pregnancy. In contrast, Cano-Ibáñez et al. [30] reported that a Mediterranean dietary pattern, based on legumes, vegetables, nuts, olive oil and whole cereals, may be related to adequate intakes of fiber, folate, vitamins D and E, calcium and iodine.

#### 3.1.3. Metabolic Aspects

The unique role of vitamins and minerals in enzymes (acting as co-factors) and transcription factors, during all stages of cell growth and differentiation, suggests that micronutrient imbalance could evoke alterations in maternal and fetal metabolism [55]. For instance, deficiencies in B-complex vitamins, which serve as coenzymes in the release of energy from macronutrients, may have an impact on cellular growth and nerve tissue development [56,57]. Furthermore, the synergistic action of methyl-donor nutrients, i.e., folate and vitamin B-12, is essential for the methylation of DNA, RNA, protein neurotransmitters and phospholipids. Hence, inadequate intake of these nutrients may increase the risk of miscarriage and fetal malformation [57,58,59].

In this context, the multifactorial benefits of the Mediterranean-type diet on pregnancy outcomes and offspring health have been well documented in a large and growing body of literature [2,3,12,14,15,23,25,60]. In fact, the superiority of this pattern is not limited to the appropriate “nutritional adequacy profile” but further expands towards a strong correlation with a low intake of proinflammatory nutrients, such as refined sugars, starches and SFA [30,53,61]. Abnormal glucose tolerance during pregnancy and long-term maternal hyperglycemia related to the consumption of starchy food items and sugar-based confectionary may result in alterations in the in utero environment, influencing fetal growth and development [62,63,64,65]. A recent study conducted by our research group [66] suggested that even small fluctuations in carbohydrate quality may be associated with significant shifts in the fetal environment that are reflected in the amniotic fluid “fingerprint”. Specifically, it was found that dietary patterns with different GIs, but similar total carbohydrate intakes, may be associated with a different glucose flux. Furthermore, different macronutrient distributions and micronutrient nutriture may induce metabolic modifications linked to amino acid metabolism and the citric acid cycle [66]; this metabolic switch may, in turn, be associated with fetal body composition, which may potentially be related to offspring’s risk of future disease [67,68].

## 4. Materials and Methods

### 4.1. Study Population

#### 4.1.1. Participants

A total of 673 women with singleton pregnancy, during the 2nd trimester, were invited to participate while visiting the 1st Department of Obstetrics and Gynecology, Papageorgiou General Hospital, Thessaloniki, Greece. All subjects gave their informed consent for inclusion before they participated in the study. The study was conducted according to the guidelines of the Declaration of Helsinki and approved by the Bioethics Committee of the Medical School, Aristotle University, Thessaloniki, Greece (A19479—26 February 2008).

#### 4.1.2. Exclusion Criteria

The exclusion criteria for the study population are described in detail in a previous publication [56]. Briefly, of the initial population (*n* = 673) enrolled in the present investigation, 48 women were excluded due to insufficient dietary information, medical complications and inconsistent answers. Furthermore, 17 participants with biologically improbable intakes (caloric intake greater than 3500 kcal per day) [69,70] were also excluded. Consequently, 608 women were finally included in the study.

### 4.2. Data Collection

#### 4.2.1. Demographic/Anthropometric and Lifestyle Characteristics

Demographic/anthropometric and lifestyle characteristics were collected through personal interviews prior to the antenatal appointment [71]. Pre-gestational body mass index was calculated as weight (in kilograms) divided by standing height (in meters) squared [72]. The physical activity status was evaluated using the short version of the International Physical Activity Questionnaire [29].

#### 4.2.2. Dietary Assessment, Nutrient Intake and Dietary Indexes

Usual maternal dietary intake was assessed using a Mediterranean-oriented, culture-specific food frequency questionnaire (FFQ), previously developed and validated for the pregnant population [71]. Data collection was conducted through personal interviews by a registered dietician or a well-trained interviewer. An appropriate Microsoft Excel database was designed for the conversion of participants’ responses into dietary data [71]. The nutrient density approach (intake per 1000 kcal) [70] was adopted in order to mitigate the dominating effect of those nutrients with the largest variance [7,22,43]. Furthermore, the ratios of carbohydrates to fiber, animal to plant protein and (MUFA + PUFA) to SFA were calculated.

In order to evaluate the dietary quality in terms of food consumption, the 221 food items of the FFQ [71] were summarized into 19 selected food groups.

Adherence to the Mediterranean diet was also evaluated using the Mediterranean Diet Score (MedDiet Score) proposed by Panagiotakos et al. [73]. Taking into account that our study population included pregnant women, we modified the index in two ways: (a) milk and dairy products were presumed to be protective components [3,23], and (b) alcohol intake was excluded, as it is not recommended during pregnancy [74]. As such, there were 10 included components (out of the initial 11 components), and given that each component is awarded a minimum score of 0 and a maximum score of 5, the potential total score ranged between 0 and 50 (the initial scale ranged between 0 and 55). Higher values were indicative of closer adherence to the Mediterranean diet [73].

The HEI-2010 total score was estimated as described by Guenther et al. [75], using the Food Patterns Equivalents Database (FPED) 2013–2014 [76]. Alcohol intake was omitted from the component of empty calories [77]. Twelve nutrient density components were summed into an overall score of a 100-point scale since no other modification was carried out [75]. Only the total score was used for further analyses. The higher the overall score, the higher the dietary quality [75].

As a measure of carbohydrate quality, the dietary GI was estimated according to Hu et al. [78]. Glycemic index values of the different foods were obtained from published international tables [79], using white bread as the standard reference.

### 4.3. Methodological and Statistical Design

**Figure 5:**
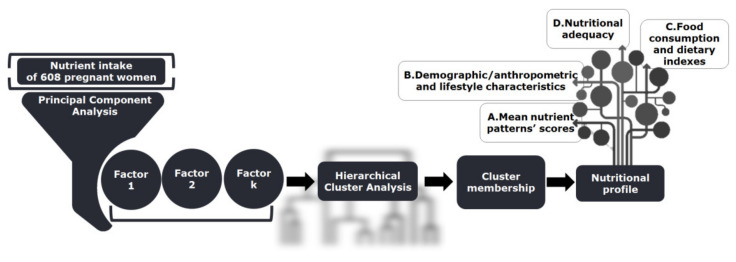
Framework of the methodology adopted in the current study.

The framework of the methodology of the current study was based on the work of Vasilopoulou et al. [80] and is illustrated briefly in Figure 5. For the extraction of nutrient patterns, PCA, with varimax rotation of the factorial axes, was performed. Hierarchical cluster analysis was conducted in order to identify homogenous groups of participants relative to nutrient patterns. The nutritional profiles of the derived groups of participants were analyzed further following a four-dimensional approach (A–D) as Figure 5 presents.

#### 4.3.1. Principal Component Analysis

Principal component analysis was based on the correlation matrix of 18 energy-adjusted nutrient variables (Figure 5). The factorability of the intercorrelation matrix was tested with the KMO index and Bartlett’s test for sphericity. Nutrient patterns were labeled and interpreted according to the nutrients with an absolute value factor loading of ≥0.5. Finally, each participant received a factor score for each nutrient pattern.

#### 4.3.2. Hierarchical Cluster Analysis

Hierarchical cluster analysis was based on the nutrient factor scores derived from the previously applied PCA analysis (Figure 5) [81,82]. Clusters were structured using Ward’s criterion [83], while the squared Euclidian distance was used as a dissimilarity index [82] between the pregnant women. Visual inspection of the dendrogram (not shown) indicated that participants were discriminated into three to six groups; however, a six-cluster structural layout was the best interpretable solution.

The contribution of each nutrient pattern in the cluster construction was evaluated by examining the magnitude and the statistical significance of the corresponding coefficients of determination *R*^2^ computed from a series of one-way ANOVAs [84]; within this statistical approach, cluster membership was considered as the independent variable, and nutrients’ pattern scores as the dependent variables. The value of *R*^2^ is indicative of the percentage of variance of the examined nutrients’ pattern scores explained by the differences between the clusters [82]. In the frame of one-way ANOVA, *R*^2^ is computationally and conceptually equivalent to the “Eta-squared” (*η*^2^) index, a measure of the independent variable’s—the cluster membership in the present study—effect size [85]. Eta-squared is calculated by the formula *η*^2^ = *R*^2^ = (SS_Between clusters_/SS_Total_), where SS indicates the corresponding sum of squares.

#### 4.3.3. Statistical Comparisons among Clusters regarding Demographic/Anthropometric Features and Dietary Quality

In order to facilitate readership, the numbering of tables as presented in the Results section is highlighted at the appropriate position herein.

Statistical comparisons among the clusters were conducted in terms of demographic/anthropometric, lifestyle and dietary characteristics (nutrient pattern scores, food consumption and dietary indexes, Figure 5A–C). Differences among clusters relative to the qualitative variables were examined using the chi-squared test (Table 2).

The homogeneity of variance among clusters was examined using Levene’s test. Parametric one-way ANOVA followed by Tukey’s test for multiple pairwise comparisons among mean values was conducted (Table 2 and Table 4, Figure 1). In the case of deviations from the homogeneity assumption, we performed Welch’s ANOVA, followed by the Games–Howell test for pairwise comparisons of mean values [86] (Figure 1).

In the case of extreme deviations of the data from the normality and homogeneity of variances, the non-parametric Kruskal–Wallis (KW) test followed by a series of pairwise Mann–Whitney (MW) tests was used to highlight cluster differences (Table 3, Figure 3). In all non-parametric tests (KW, MW) and the chi-squared test, the observed significance level (*p*-value) was computed with the Monte Carlo simulation method, utilizing 10,000 random samples. This method leads to safe inferential conclusions even in cases where the methodological assumptions of the previously mentioned tests are not satisfied.

The significance level in all hypothesis testing procedures was predetermined at α = 0.05 (*p* ≤ 0.05). Quantitative data are presented as means and SD values, while qualitative data are presented as percentages. For food intakes and dietary indexes, median values are also provided. All statistical analyses were performed using IBM SPSS v.27.0 (SPSS Inc., Chicago, IL, USA) enhanced with the module Exact Tests (for the implementation of the Monte Carlo simulation method).

#### 4.3.4. Appraisal of Nutritional Adequacy

Nutritional adequacy (Figure 5) was estimated for the following 17 nutrients: protein, carbohydrate, fiber, thiamin, riboflavin, niacin, vitamin B-6, folate, vitamins B-12 and C, calcium, phosphorus, magnesium, potassium, zinc, copper and selenium (Supplementary Table S2). For the assessment of nutritional adequacy, the Dietary Reference Intakes (DRIs) values proposed by the Institute of Medicine (IOM) were used [87,88,89,90]. Normal distribution was checked using skewness and kurtosis. All nutrients satisfied the assumption of normality in each cluster [90,91].

The appraisal of nutritional adequacy was performed using the probability approach or, where this was not applicable, the EAR cut-point method.

##### Probability Approach

In the case of nutrients with an established EAR and SD of the requirement, the probability approach proposed by Beaton was evaluated [92]. Specifically, the probability approach was performed on the following nutrients: protein, carbohydrate, thiamin, riboflavin, niacin, vitamin B-6, folate, vitamins B-12 and C, phosphorus, magnesium, zinc, copper and selenium. Calcium was excluded due to the absence of the SD of the requirement [88,90] (Supplementary Table S2).

To assess the probability of adequacy for the usual intake of each nutrient, the NORM.DIST function of MS Excel was applied, using the EAR and the SD of the requirement as parameters [90]. The adequacy of the participants in each cluster was calculated as the average of individual probabilities and expressed as a percentage (%) [89,93].

##### EAR Cut-Point Method

For nutrients with an established AI, i.e., fiber and potassium, the nutritional adequacy was estimated using the EAR cut-point method [94]. The adequacy of calcium was also assessed using this method (Supplementary Table S2).

Usual intake values for the aforementioned nutrients were taken as whole numbers or rounded to the appropriate decimals, depending on the AI or EAR values. The level of adequacy was measured, calculating the proportion of individuals with intakes above the AI/EAR.

##### Interval Estimates

In order to provide a better estimation of actual adequacy, interval estimations on the final estimates were calculated by the application of the bootstrap method [56,95,96]. As such, 95% BCa bootstrap CIs [96] were calculated around the final estimates of the probability approach and the EAR cut-point method. Each bootstrap run was based on 500 resampling circles [56].

## 5. Conclusions

The assessment of nutritional status across different populations is important for the development of public health strategies and preventive actions.

The aim of the present study was to assess the link between a posteriori dietary patterns and nutritional adequacy. Towards obtaining accurate, high-quality and reliable dietary data, in the present study, we: (a) adopted a Mediterranean-oriented semi-quantitative FFQ, developed by our research group and validated for the pregnant population; (b) accomplished the dietary recording via personal interviews by a registered dietician or a well-trained interviewer and used the “precise frequency” version [71]; (c) recruited 608 participants, a sample size large enough to further strengthen our findings.

**Figure 6:**
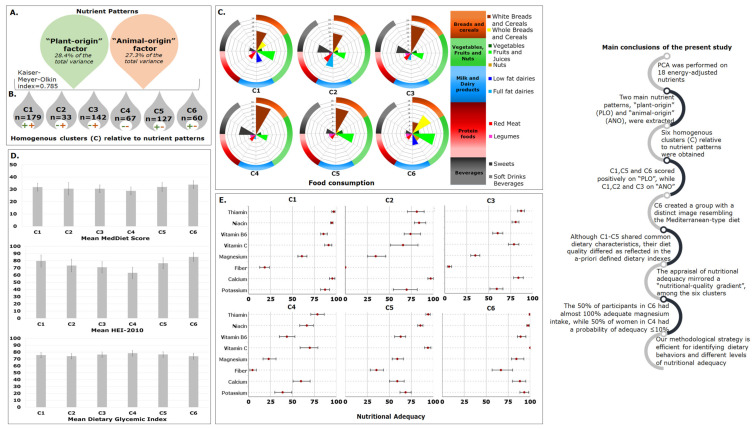
Summarized conclusions of the present study. Principal component analysis (PCA) (**A**). Hierarchical cluster analysis (HCA) (**B**). Dietary quality in terms of food choices (**C**) and dietary indexes (**D**). Appraisal of nutritional adequacy (**E**).

Based on the proposed framework, the application of PCA revealed two distinct patterns. Relative to these patterns, the 608 pregnant women were classified into six homogenous clusters. Among them, a group with a distinct image resembling that of the Mediterranean-type diet (C6, Figure 6) was unveiled when dietary quality was reflected in food choices and a priori indexes. For the realization of our ultimate goal, a robust methodology to identify “hidden” high-risk groups of participants was implemented [56]. As such, the appraisal of nutritional adequacy indicated that participants in C6 (Figure 6) were more likely to have adequate nutrient intakes compared to participants in the other clusters.

Considering pregnancy as a unique window of opportunity to promote balanced dietary behaviors and improve both maternal and offspring health, the insights gained from this study may be of assistance to the design and implementation of public health strategies well suited to the pregnant population. 

At this point, it should be emphasized that, although the current study was conducted on pregnant women, the proposed framework of the methodology could be applied to several different population groups.

## Figures and Tables

**Table 1:** Rotated factor loading matrix and explained variances for the two major nutrient patterns identified by PCA.

	Plant-Origin Factor	Animal-Origin Factor
Folate	0.858	
Magnesium	0.789	
Potasium	0.718	
Carbohydrates/Fiber	−0.707	
Thiamin	0.698	
Vitamin B-6	0.613	
Copper	0.584	
Niacin	0.545	
Vitamin C	0.527	
Phosphorus		0.813
Vitamin B-12		0.811
Animal Protein/Plant Protein		0.772
Calcium		0.753
Riboflavin		0.726
Zinc		0.652
(MUFA + PUFA)/SFA		−0.622
Selenium		0.597
Cholesterol		0.581
Variance explained (%)	28.4	27.3
Eigenvalues	5.119	4.909

For simplicity, absolute values of <0.5 are not shown in the table. PCA: principal component analysis; MUFA: monounsaturated fatty acids; PUFA: polyunsaturated fatty acids; SFA: saturated fatty acids.

**Table 2:** Demographic/anthropometric and lifestyle characteristics of participants among the six clusters.

	C1 (*n* = 179)	C2 (*n* = 33)	C3 (*n* = 142)	C4 (*n* = 67)	C5 (*n* = 127)	C6 (*n* = 60)	*p*-Value
	Mean (SD)	Mean (SD)	Mean (SD)	Mean (SD)	Mean (SD)	Mean (SD)	
Maternal age (year)	36.7 (3.6)	35.9 (3.7)	36.4 (3.6)	36.2 (4.9)	36.7 (3.5)	36.4 (3.9)	0.864
Pre-pregnancy BMI	23.5 (3.6)	23.8 (5.2)	24.1 (5.2)	23.8 (5)	24.0 (4.4)	23.7 (4.3)	0.889
	***n* (%)**	***n* (%)**	***n* (%)**	***n* (%)**	***n* (%)**	***n* (%)**	
**Education**							
>12 years	86 (48.0)	15 (45.5)	76 (53.5)	34 (50.7)	68 (53.5)	25 (41.7)	0.614
≤12 years	93 (52.0)	18 (54.5)	66 (46.5)	33 (49.3)	59 (46.5)	35 (58.3)	
Physical activity level *							
Low activity	127 (70.9)	27 (81.8)	112 (78.9)	60 (89.6)	101 (79.5)	46 (76.7)	0.194
Moderate activity	39 (21.8)	5 (15.2)	23 (16.2)	3 (4.5)	21 (16.5)	10 (16.7)	
High activity	13 (7.3)	1 (3.0)	7 (4.9)	4 (6.0)	5 (3.9)	4 (6.7)	
**Smoking during pregnancy**							
Occasional or daily smokers	20 (11.2)	5 (15.2)	27 (19.0)	15 (22.4)	21 (16.5)	3 (5.0)	0.039
Non-smokers	159 (88.8)	28 (84.8)	115 (81.0)	52 (77.6)	106 (83.5)	57 (95.0)	

C: clusters; SD: standard deviation; BMI: body mass index. * As derived from the International Physical Activity Questionnaire [29]. One-way Analysis of variance (ANOVA) was used for continuous variables; the chi-squared test was used for categorical variables.

**Table 3:** Selected food groups as percentages of total energy intake among the six clusters (C1–C6, *n* = 608).

	C1 (*n* = 179)	C2 (*n* = 33)	C3 (*n* = 142)	C4 (*n* = 67)	C5 (*n* = 127)	C6 (*n* = 60)	*p*-Value
	Median (IQR)	Median (IQR)	Median (IQR)	Median (IQR)	Median (IQR)	Median (IQR)	
White breads and cereals	9.9 (2–15.5)	9.5 (1.1–13.6)	13.6 (9.3–16.7)	14.5 (11.3–16.7)	12.5 (1.9–16.9)	5.7 (0.6–14.4)	<0.001
Whole breads and cereals	5.5 (0.4–13.1)	3.4 (0.0–10.9)	0.7 (0.0–4.4)	0.0 (0.0–0.6)	2.1 (0–11.1)	10.4 (4.9–15.4)	<0.001
Pasta, rice and potatoes	6.6 (5.2–8.2)	7 (5.6–9.1)	8.0 (6.3–9.4)	7.3 (6.3–9.1)	7.3 (5.8–8.6)	6.8 (4.4–8.2)	<0.001
Vegetables	2.9 (2.2–3.5)	2.6 (1.8–3.9)	2.9 (2.1–3.7)	2.7 (2.1–3.8)	3.5 (2.9–4.7)	3.4 (2.5–4.6)	<0.001
Fruits and juices	9.2 (6.2–12.1)	6.3 (3.6–8.4)	6.2 (3.9–8.6)	5.8 (3.7–8.6)	8.9 (6.1–12.2)	11.1 (8.1–15.2)	<0.001
Nuts	1.2 (0.0–3.5)	0.0 (0.0–1.1)	0.6 (0.0–2.2)	0.0 (0.0–2.0)	1.4 (0.0–4.3)	4.1 (0.1–8.4)	<0.001
Low-fat dairy	5.6 (0.0–8.6)	0.0 (0.0–8.0)	0.8 (0.0–5.1)	0.0 (0.0–0.0)	0.6 (0.0–4.3)	5.7 (2.6–8.0)	<0.001
Full-fat dairy	0.0 (0.0–7.5)	7.2 (0.0–15.4)	3.4 (0.0–8.3)	2.2 (0.0–6.0)	0.0 (0.0–4.3)	0.0 (0.0–3.2)	<0.001
White cheese “feta”	6.6 (3.4–8.1)	7.2 (3.7–11.5)	5.5 (3.3–7.3)	3.3 (2.6–6.5)	3.4 (1.1–6.1)	3.3 (1.1–4.9)	<0.001
Yellow cheese	2.4 (1.1–3.9)	2.5 (1.7–5.5)	2.2 (1.4–4.1)	2.0 (0.8–3.6)	1.9 (0.7–2.6)	2.0 (1.0–3.2)	0.007
Red meat	4.5 (3.2–6.1)	5.3 (3.4–7.7)	4.1 (2.9–5.3)	3.6 (2.3–5.0)	3.2 (2.2–4.3)	3.4 (2.5–4.8)	<0.001
Meat products	0.5 (0.0–1.0)	0.2 (0.0–1.6)	0.5 (0.0–0.9)	0.4 (0.0–0.7)	0.4 (0.0–0.8)	0.1 (0.0–0.6)	0.282
Poultry	1.9 (1.6–2.5)	2.0 (1.1–2.8)	1.8 (1.4–2.7)	1.7 (1.1–2.4)	1.7 (1.2–2.3)	1.8 (1.3–2.4)	0.383
Egg	0.5 (0.2–1.4)	0.5 (0.3–1.6)	0.5 (0.2–1.4)	0.2 (0.0–0.5)	0.4 (0.0–1.1)	0.4 (0.0–1.4)	<0.001
Seafood	2.4 (1.4–3.4)	2.5 (1.4–4.1)	1.9 (1.2–2.9)	1.3 (0.0–2.2)	1.6 (0.8–2.5)	2.3 (1.4–3.0)	<0.001
Legumes	2.5 (1.6–3.4)	2.0 (0.0–3.1)	2.9 (1.9–3.6)	2.8 (2.1–3.9)	3.4 (2.5–4.6)	3.3 (2.3–5.0)	<0.001
Sweets	3.6 (1.6–7.7)	7.7 (1.4–8.8)	7.3 (4.6–9.6)	8.3 (4.0–12.0)	4.8 (2.4–7.7)	3.4 (1.4–5.3)	<0.001
Soft drink beverages	0.0 (0.0–0.5)	0.0 (0.0–0.5)	0.0 (0.0–1.0)	0.4 (0.0–2.9)	0.0 (0.0–0.9)	0.0 (0.0–0.1)	0.002
“Ready-to-eat”	1.5 (0.8–1.8)	1.6 (0.0–2.8)	1.5 (0.0–3.0)	1.6 (1.3–3.2)	1.5 (0.0–2.7)	1.1 (0.0–1.6)	0.002

IQR: interquartile range, i.e., 25th percentile value (lower quartile)–75th percentile value (upper quartile). The Kruskal–Wallis test was used to test the differences in distributions among clusters.

**Table 4:** Dietary indexes among the six clusters (C1–C6, *n* = 608).

	MedDiet Score			HEI-2010			Dietary GI		
	Median	Mean (SD)	*p*-Value	Median	Mean (SD)	*p*-Value	Median	Mean (SD)	*p*-Value
**C1**	32.0	31.8 (3.2) ^b^	<0.001	79.6	79.7 (8.4) ^b^	<0.001	76.0	75.6 (3.9) ^b,c^	<0.001
**C2**	31.0	30.6 (5.1) ^b^		72.8	73.3 (8.9) ^c,d^		73.8	74.1 (4.0) ^c^	
**C3**	30.0	30.5 (3.1) ^b^		71.2	70.9 (7.9) ^d^		76.4	76.4 (3.9) ^b^	
**C4**	29.0	28.7 (3.2) ^c^		63.7	63.2 (8.0) ^e^		78.6	78.5 (4.2) ^a^	
**C5**	32.0	31.9 (3.7) ^b^		75.8	76.6 (7.6) ^b,c^		76.7	76.6 (4.0) ^a,b^	
**C6**	34.0	33.8 (3.3) ^a^		86.0	85.2 (6.3) ^a^		74.6	74.0 (4.4) ^c^	

HEI-2010: Healthy Eating Index 2010; GI: dietary glycemic index; SD: standard deviation. Different superscript letters represent statistically significant differences at *p* < 0.05, according to one-way ANOVA and Tukey’s test.

**Table 5:** Percentile distribution of the probability of adequacy for selected micronutrients.

	Magnesium					Zinc					Copper				
	*P10*	*P25*	*P50*	*P75*	*P90*	*P10*	*P25*	*P50*	*P75*	*P90*	*P10*	*P25*	*P50*	*P75*	*P90*
**C1**	12	32	65	95	100	90	98	100	100	100	99	100	100	100	100
**C2**	1	8	28	60	83	85	96	100	100	100	53	84	98	100	100
**C3**	1	4	17	60	96	67	81	95	100	100	93	98	100	100	100
**C4**	0	2	10	34	79	12	40	77	97	100	80	98	100	100	100
**C5**	5	19	69	95	100	30	62	90	99	100	100	100	100	100	100
**C6**	34	78	97	100	100	83	97	100	100	100	100	100	100	100	100
	**Selenium**					**Thiamin**					**Riboflavin**				
	***P10***	***P25***	***P50***	***P75***	***P90***	***P10***	***P25***	***P50***	***P75***	***P90***	***P10***	***P25***	***P50***	***P75***	***P90***
**C1**	100	100	100	100	100	96	100	100	100	100	100	100	100	100	100
**C2**	100	100	100	100	100	38	69	99	100	100	100	100	100	100	100
**C3**	98	100	100	100	100	48	88	99	100	100	98	100	100	100	100
**C4**	39	87	100	100	100	27	56	97	100	100	25	77	99	100	100
**C5**	54	97	100	100	100	74	99	100	100	100	40	95	100	100	100
**C6**	97	99	100	100	100	100	100	100	100	100	100	100	100	100	100
	**Niacin**					**Vitamin B-6**					**Vitamin C**				
	***P10***	***P25***	***P50***	***P75***	***P90***	***P10***	***P25***	***P50***	***P75***	***P90***	***P10***	***P25***	***P50***	***P75***	***P90***
**C1**	82	95	99	100	100	38	88	98	100	100	68	100	100	100	100
**C2**	51	66	95	99	100	20	59	83	99	100	0	1	100	100	100
**C3**	44	71	90	98	100	17	32	63	93	100	3	76	100	100	100
**C4**	13	32	80	94	99	2	7	35	81	99	0	35	100	100	100
**C5**	56	79	93	99	100	11	32	73	99	100	98	100	100	100	100
**C6**	91	99	100	100	100	65	82	99	100	100	100	100	100	100	100

*P*: percentile; k-percentile is the k% of individuals in each cluster that is below the respective probability of adequacy, e.g., in the case of magnesium, almost 18 participants in C1 (*P*10) had a probability of adequacy below 12%; C: cluster; number of participants in each cluster: C1 (*n* = 179), C2 (*n* = 33), C3 (*n* = 142), C4 (*n* = 67), C5 (*n* = 127), C6 (*n* = 60).

**Table 6:** Percentile distribution of usual intake and percentage of “adequate” population for fiber, potassium and calcium.

	Fiber Intake (*AI* = 28 g/d)					Percentage of “Adequate” Population *
	*P10*	*P25*	*P50*	*P75*	*P90*	
**C1**	18	21	24	26	29	18.4
**C2**	11	16	17	19	24	0.0
**C3**	15	18	20	22	25	4.9
**C4**	16	17	20	23	25	4.5
**C5**	20	22	25	30	33	35.4
**C6**	24	27	30	33	38	66.7
	**Potassium Intake (*AI* = 2.9 g/d)**					
	***P10***	***P25***	***P50***	***P75***	***P90***	
**C1**	2.8	3.0	3.3	3.6	3.9	87.2
**C2**	2.5	2.7	3.0	3.3	3.5	69.7
**C3**	2.4	2.7	2.9	3.3	3.6	59.2
**C4**	2.3	2.4	2.7	3.0	3.3	38.8
**C5**	2.6	2.8	3.2	3.6	3.9	68.5
**C6**	2.9	3.1	3.5	3.8	4.2	93.3
	**Calcium Intake (*EAR* = 800 mg/d)**					
	***P10***	***P25***	***P50***	***P75***	***P90***	
**C1**	884	985	1134	1274	1441	95.5
**C2**	908	1108	1272	1492	1657	97.0
**C3**	718	868	1005	1127	1303	83.8
**C4**	600	701	832	947	1038	59.7
**C5**	570	724	839	1018	1170	59.1
**C6**	760	906	983	1146	1297	88.3

AI: adequate intake; * percentage of population with intakes above the estimated average requirement (EAR)/AI; *P*: percentile; k-percentile is the k% of individuals in each cluster that are below the respective nutrient intake, e.g., in the case of potassium, almost 18 participants in C1 (*P*10) had an intake below 2.8 g/d; C: cluster; number of participants in each cluster: C1 (*n* = 179), C2 (*n* = 33), C3 (*n* = 142), C4 (*n* = 67), C5 (*n* = 127), C6 (*n* = 60).

## Data Availability

Data are contained within the article or Supplementary Materials.

## Supplementary Materials

The following supporting information can be downloaded at: https://www.mdpi.com/article/10.3390/metabo12050395/s1. Figure S1: Mean intake of nutrients with absolute factor score ≥ 0.5 in “Plant origin” factor, among the six clusters (C1–C6) and in total sample; Figure S2: Mean intake of nutrients with absolute factor score ≥ 0.5 in “Animal origin” factor, across the six clusters (C1–C6) and in total sample; Figure S3: Comparison of energy contribution of selected food groups across the six clusters (C1–C6); Statistical significance was assessed at α = 0.05 (*p* ≤ 0.05) using the Mann–Whitney U test; Table S1: Percentile distribution of probability of adequacy for macronutrients, phosphorus, folate and vitamin B-12; Table S2: Applied methods for estimating the nutrient adequacy for the macro- and micronutrients under study.

## References

[1] Koletzko B., Brands B., Poston L., Godfrey K., Demmelmair H. (2012). Symposium on ‘Metabolic flexibility in animal and human nutrition’ Session I: Early nutrition programming, life performance and cognitive function: Early nutrition programming of long-term health. Proc. Nutr. Soc..

[2] Lecorguillé M., Teo S., Phillips C.M. (2021). Maternal Dietary Quality and Dietary Inflammation Associations with Offspring Growth, Placental Development, and DNA Methylation. Nutrients.

[3] Gesteiro E., Sánchez-Muniz F.J., Bastida S., Preedy V.R., Ross Watson R. (2020). Mediterranean diet and pregnancy. The Mediterranean Diet.

[4] Cetin I., Bühling K., Demir C., Kortam A., Prescott S.L., Yamashiro Y., Yarmolinskaya M., Koletzko B. (2019). Impact of micronutrient status during pregnancy on early nutrition programming. Ann. Nutr. Metab..

[5] Barker D.J. (2012). Developmental origins of chronic disease. Public Health.

[6] Koletzko B., Brands B., Chourdakis M., Cramer S., Grote V., Hellmuth C., Kirchberg F., Prell C., Rzehak P., Uhl O. (2014). The Power of Programming and the EarlyNutrition project: Opportunities for health promotion by nutrition during the first thousand days of life and beyond. Ann. Nutr. Metab..

[7] Conradie C., Baumgartner J., Malan L., Symington E.A., Cockeran M., Smuts C.M., Faber M. (2021). A priori and a posteriori dietary patterns among pregnant women in Johannesburg: South Africa: The nuped study. Nutrients.

[8] Parisi F., Laoreti A., Cetin I. (2014). Multiple micronutrient needs in pregnancy in industrialized countries. Ann. Nutr. Metab..

[9] Hanson M.A., Bardsley A., De-Regil L.M., Moore S.E., Oken E., Poston L., Ma R.C., McAuliffe F.M., Maleta K., Chittaranjan N. (2015). The International Federation of Gynecology and Obstetrics (FIGO) recommendations on adolescent, preconception, and maternal nutrition: “Think Nutrition First”. Int. J. Gynaecol. Obstet..

[10] Blumfield M.L., Hure A.J., Macdonald-Wicks L., Smith R., Collins C.E. (2013). Micronutrient intakes during pregnancy in developed countries: Systematic review and meta-analysis. Nutr. Rev..

[11] Dhonukshe-Rutten R.A., Bouwman J., Brown K.A., Cavelaars A.E., Collings R., Grammatikaki E., de Groot L.C., Gurinovic M., Harvey L.J., Hermoso M. (2013). EURRECA—Evidence-based methodology for deriving micronutrient recommendations. Crit. Rev. Food Sci. Nutr..

[12] Amati F., Hassounah S., Swaka A. (2019). The impact of Mediterranean dietary patterns during pregnancy on maternal and offspring health. Nutrients.

[13] Ancira-Moreno M., O’Neill M.S., Rivera-Dommarco J.Á., Batis C., Rodríguez Ramírez S., Sánchez B.N., Castillo-Castrejón M., Vadillo-Ortega F. (2020). Dietary patterns and diet quality during pregnancy and low birthweight: The PRINCESA cohort. Matern. Child. Nutr..

[14] Raghavan R., Dreibelbis C., Kingshipp B.L., Wong Y.P., Abrams B., Gernand A.D., Rasmussen K.M., Siega-Riz A.M., Stang J., Casavale K.O. (2019). Dietary patterns before and during pregnancy and maternal outcomes: A systematic review. Am. J. Clin. Nutr..

[15] Chen X., Zhao D., Mao X., Xia Y., Baker P.N., Zhang H. (2016). Maternal dietary patterns and pregnancy outcome. Nutrients.

[16] Lu M.S., Chen Q.Z., He J.R., Wei X.L., Lu J.H., Li S.H., Wen X.X., Chan F.F., Chen N.N., Qiu L. (2016). Maternal dietary patterns and fetal growth: A large prospective cohort study in China. Nutrients.

[17] Coelho N.D., Cunha D.B., Esteves A.P., Lacerda E.M., Theme M.M. (2015). Dietary patterns in pregnancy and birth weight. Rev. Saude Publica.

[18] Chia A.R., Chen L.W., Lai J.S., Wong C.H., Neelakantan N., van Dam R.M., Chong M.F. (2019). Maternal dietary patterns and birth outcomes: A systematic review and meta-analysis. Adv. Nutr..

[19] Colón-Ramos U., Racette S.B., Ganiban J., Nguyen T.G., Kocak M., Carroll K.N., Völgyi E., Tylavsky F.A. (2015). Association between dietary patterns during pregnancy and birth size measures in a diverse population in Southern US. Nutrients.

[20] Lovegrove J.A., Hodson L., Sharma S., Lanham-New S.A. (2015). Nutrition Research Methodologies.

[21] Martin C.L., Sotres-Alvarez D., Siega-Riz A.M. (2015). Maternal dietary patterns during the second trimester are associated with preterm birth. J. Nutr..

[22] Moskal A., Pisa P.T., Ferrari P., Byrnes G., Freisling H., Boutron-Ruault M.C., Cadeau C., Nailler L., Wendt A., Kühn T. (2014). Nutrient patterns and their food sources in an International Study Setting: Report from the EPIC study. PLoS ONE.

[23] Gete D.G., Waller M., Mishra G.D. (2020). Effects of maternal diets on preterm birth and low birth weight: A systematic review. Br. J. Nutr..

[24] Abdollahi S., Soltani S., de Souza R.J., Forbes S.C., Toupchian O., Salehi-Abargouei A. (2021). Associations between maternal dietary patterns and perinatal outcomes: A systematic review and meta-analysis of cohort studies. Adv. Nutr..

[25] Biagi C., Di Nunzio M., Bordoni A., Gori D., Lanari M. (2019). Effect of adherence to Mediterranean diet during pregnancy on children’s health: A systematic review. Nutrients.

[26] Doyle I.M., Borrmann B., Grosser A., Razum O., Spallek J. (2017). Determinants of dietary patterns and diet quality during pregnancy: A systematic review with narrative synthesis. Public Health Nutr..

[27] McGowan C.A., McAuliffe F.M. (2013). Maternal dietary patterns and associated nutrient intakes during each trimester of pregnancy. Public Health Nutr..

[28] Okubo H., Miyake Y., Sasaki S., Tanaka K., Murakami K., Hirota Y., Child Health Study Group (2011). Nutritional adequacy of three dietary patterns defined by cluster analysis in 997 pregnant Japanese women: The Osaka Maternal and Child Health Study. Public Health Nutr..

[29] International Physical Activity Questionnaire (IPAQ). The IPAQ Home Page. https://sites.google.com/site/theipaq/home.

[30] Cano-Ibáñez N., Martínez-Galiano J.M., Luque-Fernández M.A., Martín-Peláez S., Bueno-Cavanillas A., Delgado-Rodríguez M. (2020). Maternal dietary patterns during pregnancy and their association with gestational weight gain and nutrient adequacy. Int. J. Environ. Res. Public Health.

[31] Fernández-Gómez E., Luque-Vara T., Moya-Fernández P.J., López-Olivares M., Gallardo-Vigil M.Á., Enrique-Mirón C. (2020). Factors influencing dietary patterns during pregnancy in a culturally diverse society. Nutrients.

[32] Mitku A.A., Zewotir T., North D., Jeena P., Naidoo R.N. (2020). The differential effect of maternal dietary patterns on quantiles of Birthweight. BMC Public Health.

[33] Miura K., Takamori A., Hamazaki K., Tsuchida A., Tanaka T., Origasa H., Inadera H., Japan Environment and Children’s Study Group (2020). Dietary patterns during pregnancy and health-related quality of life: The Japan Environment and Children’s Study. PLoS ONE.

[34] Zhao J., Li Z., Gao Q., Zhao H., Chen S., Huang L., Wang W., Wang T. (2021). A review of statistical methods for dietary pattern analysis. Nutr. J..

[35] Edefonti V., De Vito R., Dalmartello M., Patel L., Salvatori A., Ferraroni M. (2020). Reproducibility and validity of a posteriori dietary patterns: A systematic review. Adv. Nutr..

[36] Shakya P.R., Melaku Y.A., Page A.J., Gill T.K. (2021). Nutrient patterns and depressive symptoms among Australian adults. Eur. J. Nutr..

[37] Gomes C.B., Malta M.B., Papini S.J., Benício M.H., Corrente J.E., Carvalhaes M.A. (2019). Adherence to dietary patterns during pregnancy and association with maternal characteristics in pregnant Brazilian women. Nutrition.

[38] Hajizadeh B., Jessri M., Akhoondan M., Moasheri S.M., Rashidkhani B. (2012). Nutrient patterns and risk of esophageal squamous cell carcinoma: A case-control study. Dis. Esophagus.

[39] Timmermans S., Steegers-Theunissen R.P., Vujkovic M., Bakker R., den Breeijen H., Raat H., Russcher H., Lindemans J., Hofman A., Jaddoe V.W. (2011). Major dietary patterns and blood pressure patterns during pregnancy: The Generation R Study. Am. J. Obstet. Gynecol..

[40] Vajdi M., Farhangi M.A., Nikniaz L. (2020). Diet-derived nutrient patterns and components of metabolic syndrome: A cross-sectional community-based study. BMC Endocr. Disord..

[41] Richard E.L., Laughlin G.A., Kritz-Silverstein D., Reas E.T., Barrett-Connor E., McEvoy L.K. (2018). Dietary patterns and cognitive function among older community-dwelling adults. Nutrients.

[42] Cao Y., Wittert G., Taylor A.W., Adams R., Appleton S., Shi Z. (2017). Nutrient patterns and chronic inflammation in a cohort of community dwelling middle-aged men. Clin. Nutr..

[43] Freisling H., Pisa P.T., Ferrari P., Byrnes G., Moskal A., Dahm C.C., Vergnaud A.C., Boutron-Ruault M.C., Fagherazzi G., Cadeau C. (2016). Main nutrient patterns are associated with prospective weight change in adults from 10 European countries. Eur. J. Nutr..

[44] Melaku Y.A., Gill T.K., Appleton S.L., Taylor A.W., Adams R., Shi Z. (2017). Prospective associations of dietary and nutrient patterns with fracture risk: A 20-year follow-up study. Nutrients.

[45] Wesołowska E., Jankowska A., Trafalska E., Kałużny P., Grzesiak M., Dominowska J., Hanke W., Calamandrei G., Polańska K. (2019). Sociodemographic, lifestyle, environmental and pregnancy-related determinants of dietary patterns during pregnancy. Int. J. Environ. Res. Public Health.

[46] Englund-Ögge L., Brantsæter A.L., Sengpiel V., Haugen M., Birgisdottir B.E., Myhre R., Meltzer H.M., Jacobsson B. (2014). Maternal dietary patterns and preterm delivery: Results from large prospective cohort study. BMJ.

[47] Garay S.M., Savory K.A., Sumption L., Penketh R., Janssen A.B., John R.M. (2019). The grown in Wales study: Examining dietary patterns, custom birthweight centiles and the risk of delivering a small-for-gestational age (SGA) infant. PLoS ONE.

[48] Völgyi E., Carroll K.N., Hare M.E., Ringwald-Smith K., Piyathilake C., Yoo W., Tylavsky F.A. (2013). Dietary patterns in pregnancy and effects on nutrient intake in the Mid-South: The Conditions Affecting Neurocognitive Development and Learning in Early Childhood (CANDLE) study. Nutrients.

[49] Karageorgou D., Magriplis E., Mitsopoulou A.V., Dimakopoulos I., Bakogianni I., Micha R., Michas G., Chourdakis M., Ntouroupi T., Tsaniklidou S.M. (2019). Dietary patterns and lifestyle characteristics in adults: Results from the Hellenic National Nutrition and Health Survey (HNNHS). Public Health.

[50] Costacou T., Bamia C., Ferrari P., Riboli E., Trichopoulos D., Trichopoulou A. (2003). Tracing the Mediterranean diet through principal components and cluster analyses in the Greek population. Eur. J. Clin. Nutr..

[51] Ni Y., Szpiro A., Loftus C., Tylavsky F., Kratz M., Bush N.R., LeWinn K.Z., Sathyanarayana S., Enquobahrie D.A., Davis R. (2021). Associations Between Maternal Nutrition in Pregnancy and Child Blood Pressure at 4–6 Years: A Prospective Study in a Community-Based Pregnancy Cohort. J. Nutr..

[52] Yang J., Dang S., Cheng Y., Qiu H., Mi B., Jiang Y., Qu P., Zeng L., Wang Q., Li Q. (2017). Dietary intakes and dietary patterns among pregnant women in Northwest China. Public Health Nutr..

[53] Grieger J.A., Grzeskowiak L.E., Clifton V.L. (2014). Preconception dietary patterns in human pregnancies are associated with preterm delivery. J. Nutr..

[54] Northstone K., Emmett P.M., Rogers I. (2008). Dietary patterns in pregnancy and associations with nutrient intakes. Br. J. Nutr..

[55] Ashworth C.J., Antipatis C. (2001). Micronutrient programming of development throughout gestation. Reproduction.

[56] Tsakoumaki F., Kyrkou C., Athanasiadis A.P., Menexes G., Michaelidou A.M. (2021). Nutritional Inadequacy: Unraveling the Methodological Challenges for the Application of the Probability Approach or the EAR Cut-Point Method—A Pregnancy Perspective. Nutrients.

[57] Gernand A.D., Schulze K.J., Stewart C.P., West K.P., Christian P. (2016). Micronutrient deficiencies in pregnancy worldwide: Health effects and prevention. Nat. Rev. Endocrinol..

[58] Mousa A., Naqash A., Lim S. (2019). Macronutrient and micronutrient intake during pregnancy: An overview of recent evidence. Nutrients.

[59] Marangoni F., Cetin I., Verduci E., Canzone G., Giovannini M., Scollo P., Corsello G., Poli A. (2016). Maternal diet and nutrient requirements in pregnancy and breastfeeding. An Italian consensus document. Nutrients.

[60] Caradonna F., Consiglio O., Luparello C., Gentile C. (2020). Science and healthy meals in the world: Nutritional epigenomics and nutrigenetics of the mediterranean diet. Nutrients.

[61] Esposito K., Maiorino M.I., Bellastella G., Panagiotakos D.B., Giugliano D. (2017). Mediterranean diet for type 2 diabetes: Cardiometabolic benefits. Endocrine.

[62] Du H., Van Der A.D.L., Feskens E.J. (2006). Dietary glycaemic index: A review of the physiological mechanisms and observed health impacts. Acta Cardiol..

[63] Ludwig D.S., Eckel R.H. (2002). The glycemic index at 20 y. Am. J. Clin. Nutr..

[64] Jenkins D.J., Kendall C.W., Augustin L.S., Franceschi S., Hamidi M., Marchie A., Jenkins A.L., Axelsen M. (2002). Glycemic index: Overview of implications in health and disease. Am. J. Clin. Nutr..

[65] Knopp R.H. (1997). Hormone-mediated Changes in Nutrient Metabolism in Pregnancy: A Physiological Basis for Normal Fetal Development. Ann. N. Y. Acad. Sci..

[66] Fotiou M., Fotakis C., Tsakoumaki F., Athanasiadou E., Kyrkou C., Dimitropoulou A., Tsiaka T., Chatziioannou A.C., Sarafidis K., Menexes G. (2018). H NMR-based metabolomics reveals the effect of maternal habitual dietary patterns on human amniotic fluid profile. Sci. Rep..

[67] Maslova E., Halldorsson T.I., Astrup A., Olsen S.F. (2015). Dietary protein-to-carbohydrate ratio and added sugar as determinants of excessive gestational weight gain: A prospective cohort study. BMJ Open.

[68] Blumfield M.L., Hure A.J., MacDonald-Wicks L.K., Smith R., Simpson S.J., Giles W.B., Raubenheimer D., Collins C.E. (2012). Dietary balance during pregnancy is associated with fetal adiposity and fat distribution. Am. J. Clin. Nutr..

[69] European Food Safety Authority (EFSA) (2013). Panel on dietetic products, nutrition and allergies (NDA), scientific opinion on dietary reference values for energy. EFSA J..

[70] Willett W.C. (1998). Nutrition Epidemiology.

[71] Athanasiadou E., Kyrkou C., Fotiou M., Tsakoumaki F., Dimitropoulou A., Polychroniadou E., Menexes G., Athanasiadis A.P., Biliaderis C.G., Michaelidou A.M. (2016). Development and validation of a Mediterranean oriented culture-specific semi-quantitative food frequency questionnaire. Nutrients.

[72] World Health Organisation (WHO) 2019 Europe, WHO Body Mass Index-BMI. http://www.euro.who.int/en/health-topics/diseaseprevention/nutrition/a-healthylifestyle/body-mass-index-bmi.

[73] Panagiotakos D.B., Pitsavos C., Stefanadis C. (2006). Dietary patterns: A Mediterranean diet score and its relation to clinical and biological markers of cardiovascular disease risk. Nutr. Metab. Cardiovasc. Dis..

[74] Papazian T., Serhal A., Hout H., Younes H., Abi Tayeh G., Azouri J., Lteif F.H., Kesrouani A., Khabbaz L.R. (2019). Discrepancies among different tools evaluating Mediterranean diet adherence during pregnancy, correlated to maternal anthropometric, dietary and biochemical characteristics. Clin. Nutr..

[75] Guenther P.M., Casavale K.O., Reedy J., Kirkpatrick S.I., Hiza H.A., Kuczynski K.J., Kahle L.L., Krebs-Smith S.M. (2013). Update of the healthy eating index: HEI-2010. J. Acad. Nutr. Diet..

[76] Bowman S.A., Clemens J.C., Friday J.E., Lynch K.L., Moshfegh A.J. (2017). Food Patterns Equivalents Database 2013–14: Methodology and User Guide. www.ars.usda.gov/Services/docs.htm?docid=23870.

[77] Zhu Y., Hedderson M.M., Sridhar S., Xu F., Feng J., Ferrara A. (2019). Poor diet quality in pregnancy is associated with increased risk of excess fetal growth: A prospective multi-racial/ethnic cohort study. Int. J. Epidemiol..

[78] Hu Y., Block G., Sternfeld B., Sowers M. (2009). Dietary glycemic load, glycemic index, and associated factors in a multiethnic cohort of midline women. JACN.

[79] Foster-Powell K., Holt S.H.A., Brand-Miller J.C. (2002). International table of glycemic index and glycemic load values: 2002. Am. J. Clin. Nutr..

[80] Vasilopoulou A., Galitsianos I., Fotiou M., Menexes G., Tsakoumaki F., Tsitlakidou P., Psirropoulos D., Michaelidou A.M. (2015). An exploratory study of dietary intake patterns among adults diagnosed with cardiovascular risk factors. Int. J. Food Sci. Nutr..

[81] Hair J.F., Black W.C., Babin B.J., Anderson R.E. (2010). Multivariate Data Analysis: A Global Perspective. Canonical Correlation: A Supplement to Multivariate Data Analysis.

[82] Sharma S. (1996). Cluster analysis. Applied Multivariate Techniques.

[83] Ward J.H. (1963). Hierarchical grouping to optimize an objective function. J. Am. Stat. Assoc..

[84] Michos M.C., Mamolos A.P., Menexes G.C., Tsatsarelis C.A., Tsirakoglou V.M., Kalburtji K.L. (2012). Energy inputs, outputs and greenhouse gas emissions in organic, integrated and conventional peach orchards. Ecol. Indic..

[85] Cortina J.M., Nouri H. (2011). Effect Size for ANOVA Designs.

[86] Toothaker L.E. (1993). Multiple Comparison Procedures.

[87] Oria M., Harrison M., Stallings V.A. (2019). Institute of Medicine (US) Committee to Review the Dietary Reference Intakes for Sodium and Potassium. Dietary Reference Intakes for Sodium and Potassium.

[88] Ross A.C., Taylor C.L., Yaktine A.L., Del Valle H.B. (2011). Institute of Medicine (US) Committee to Review Dietary Reference Intakes for Vitamin D and Calcium. Dietary Reference Intakes for Calcium and Vitamin D.

[89] IOM 2000 Institute of Medicine (US) Standing Committee on the Scientific Evaluation of Dietary Reference Intakes (2000). Dietary Reference Intakes: Applications in Dietary Assessment.

[90] Otten J.J., Pitzi Hellwig J., Meyers L.D. (2006). Dietary Reference Intakes: The Essential Guide to Nutrient Requirements.

[91] Murphy S.P., Poos M.I. (2002). Dietary reference intakes: Summary of applications in dietary assessment. Public Health Nutr..

[92] Beaton G.H. (1985). Uses and limits of the use of the Recommended Dietary Allowances for evaluating dietary intake data. Am. J. Clin. Nutr..

[93] Murphy S.P., Vorster H.H. (2007). Methods for using nutrient intake values (NIVs) to assess or plan nutrient intakes. Food Nutr. Bull..

[94] Bailey R.L., Pac S.G., Fulgoni V.L., Reidy K.C., Catalano P.M. (2019). Estimation of total usual dietary intakes of pregnant women in the United States. JAMA Netw. Open.

[95] European Food Safety Authority (EFSA) (2012). EFSA Panel on Plant Protection Products and their Residues (PPR). Guidance on the use of probabilistic methodology for modelling dietary exposure to pesticide residues. EFSA J..

[96] Efron B. (1987). Better bootstrap confidence intervals. JASA.

