# Supplementary material for: Framework of Methodology to Assess the Link between A Posteriori Dietary Patterns and Nutritional Adequacy: Application to Pregnancy
Source: Metabolites. 2022 Apr 27;12(5):395. doi: 10.3390/metabo12050395 (PMC9148035; doi:10.3390/metabo12050395)
Supplement: Supplementary file 1 [file metabolites-12-00395-s001.zip › Metabolite templates-Supplementary Material -Tsakoumaki et al..pdf]

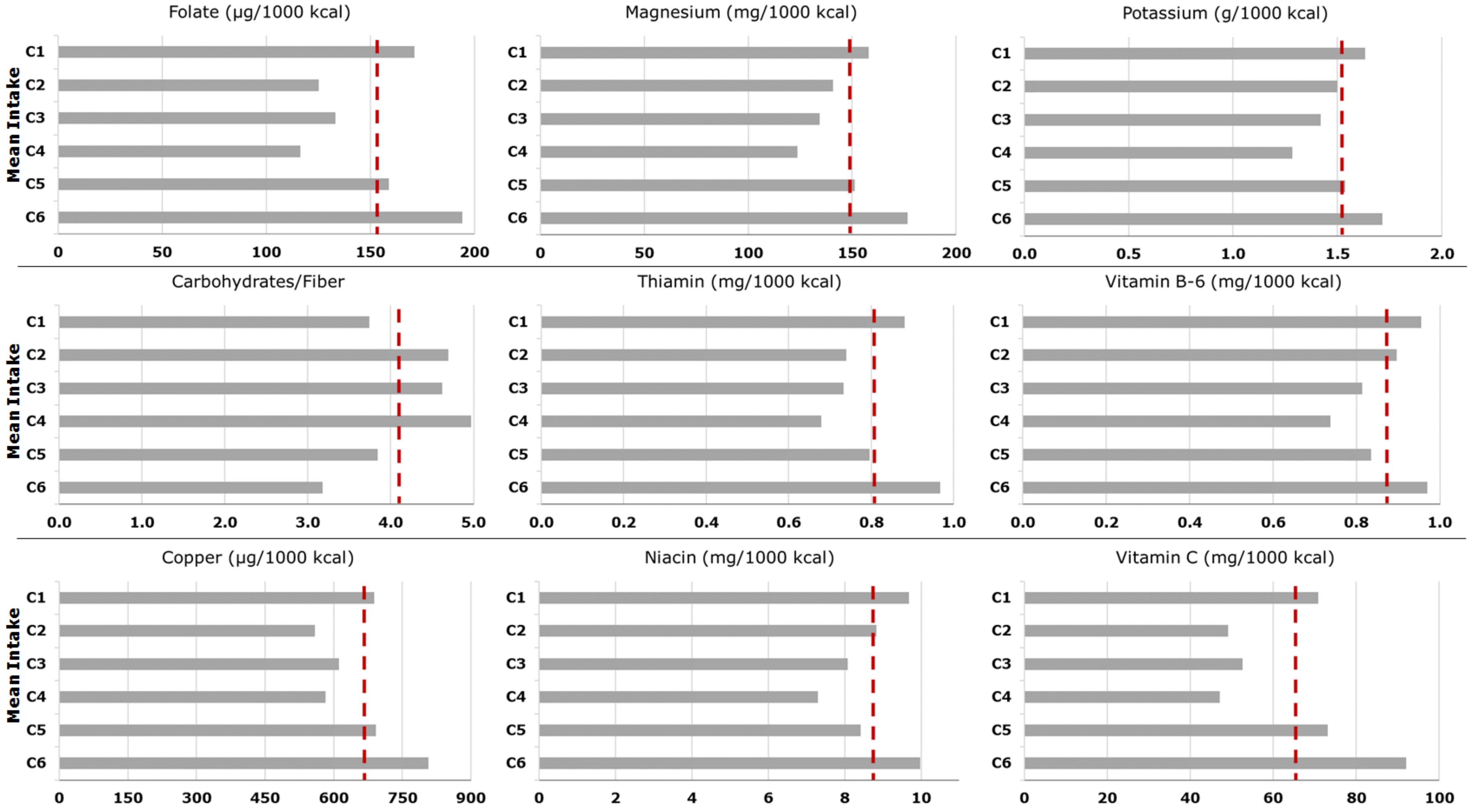

Supplementary Figure S1. Mean intake of nutrients with absolute factor score ≥0.5 in "Plant origin" factor, among the six clusters (C1-C6) and in total sample ( ).

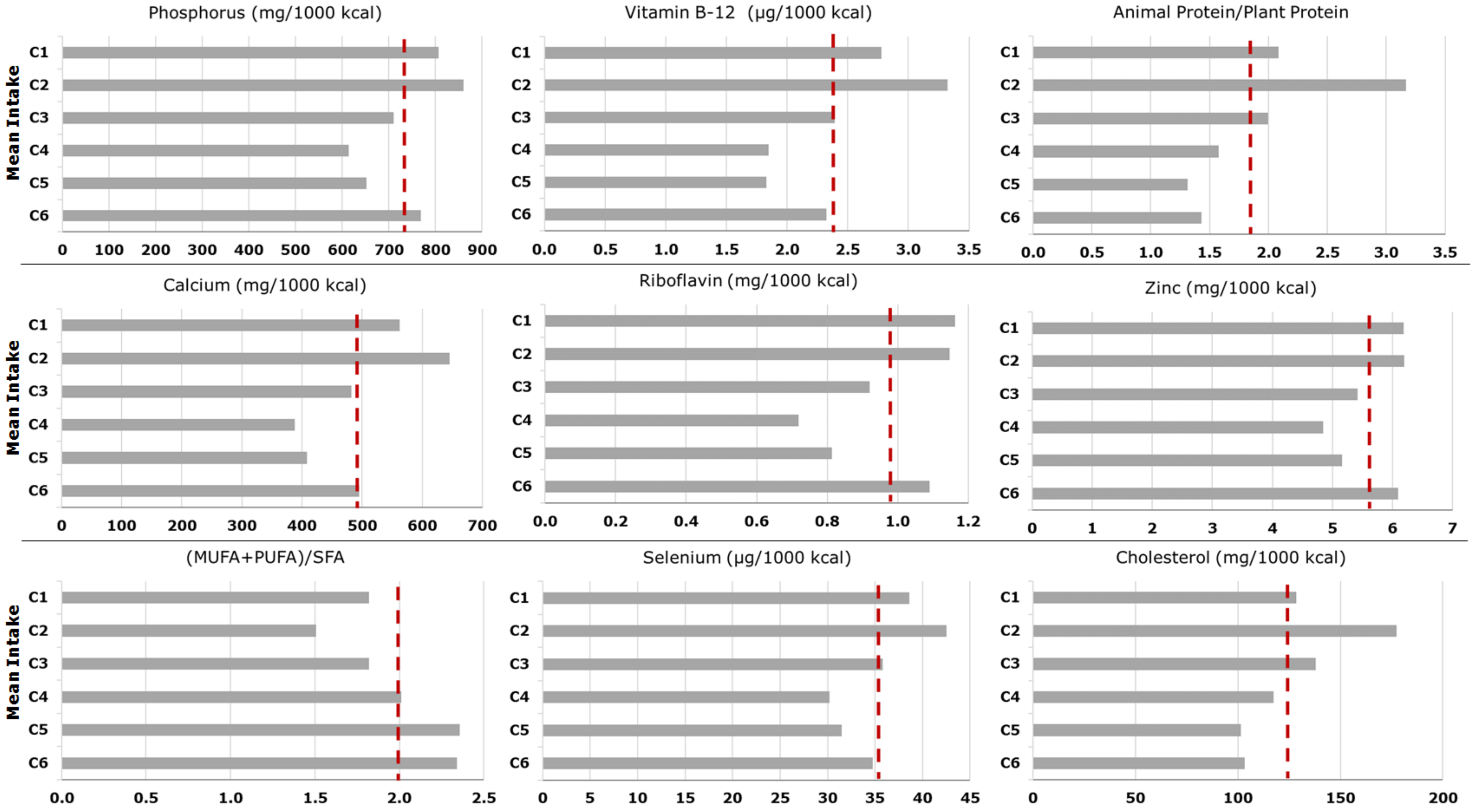

Supplementary Figure S2. Mean intake of nutrients with absolute factor score ≥0.5 in “Animal origin” factor, across the six clusters (C1-C6) and in total sample (▬ ▬ ▪). MUFA: Monounsaturated fatty acids; PUFA: Polyunsaturated fatty acids; SFA: Saturated fatty acids.

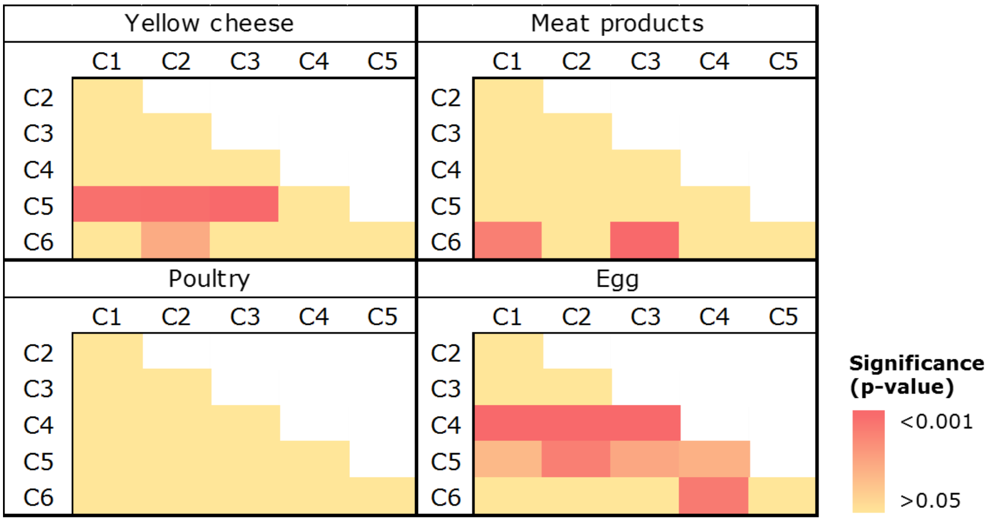

Supplementary Figure S3. Comparison of energy contribution of selected food groups across the six clusters (C1-C6); Statistical significance was assessed at α=0.05 (p≤0.05) using the Mann-Whitney U test.

Supplementary Table S1. Percentile distribution of probability of adequacy for macronutrients, phosphorus, folate and vitamin B-12

	**Protein**					**Carbohydrate**				
	P10	***P25***	***P50***	***P75***	***P90***	***P10***	***P25***	***P50***	***P75***	***P90***
**C1**	95	100	100	100	100	98	100	100	100	100
**C2**	100	100	100	100	100	83	94	99	100	100
**C3**	79	100	100	100	100	99	100	100	100	100
**C4**	59	99	100	100	100	100	100	100	100	100
**C5**	81	99	100	100	100	100	100	100	100	100
**C6**	93	100	100	100	100	100	100	100	100	100
	**Phosphorus**					**Folate**				
	P10	***P25***	***P50***	***P75***	***P90***	***P10***	***P25***	***P50***	***P75***	***P90***
**C1**	100	100	100	100	100	0	0	0	0	3
**C2**	100	100	100	100	100	0	0	0	0	0
**C3**	100	100	100	100	100	0	0	0	0	0
**C4**	100	100	100	100	100	0	0	0	0	0
**C5**	100	100	100	100	100	0	0	0	0	3
**C6**	100	100	100	100	100	0	0	1	6	33
	**Vitamin B-12**									
	P10	***P25***	***P50***	***P75***	***P90***					
**C1**	100	100	100	100	100					
**C2**	100	100	100	100	100					
**C3**	100	100	100	100	100					
**C4**	99	100	100	100	100					
**C5**	92	100	100	100	100					
**C6**	100	100	100	100	100					

P: Percentile; k-percentile is the k% of individuals in each cluster that is below to the respective probability of adequacy, e.g. in the case of protein, almost 18 participants in C1 (*P*10) had a probability of adequacy below 95%; C: Cluster; Number of participants in each cluster: C1 (n=179), C2 (n=33), C3 (n=142), C4 (n=67), C5 (n=127), C6 (n=60).

Supplementary Table S2. Applied methods for estimating the nutrient adequacy for the macro- and micronutrients under study.

		Probability approach	EAR cut-point method	Comments
1	**Protein**	+		
2	**Carbohydrate**	+		
3	**Fiber**		+	Established AI
4	**Thiamin**	+		
5	**Rivoflavin**	+		
6	**Niacin**	+		
7	**Vitamin B-6**	+		
8	**Folate**	+		
9	**Vitamin B-12**	+		
10	**Vitamin C**	+		
11	**Calcium**		+	Not established SD
12	**Phosphorus**	+		
13	**Magnesium**	+		
14	**Potassium**		+	Established AI
15	**Zinc**	+		
16	**Copper**	+		
17	**Selenium**	+		

AI: Adequate Intake; SD: standard deviation
